# Transforaminal Approach in Thoracal Disc Pathologies: Transforaminal Microdiscectomy Technique

**DOI:** 10.1155/2014/301945

**Published:** 2014-04-15

**Authors:** Sedat Dalbayrak, Onur Yaman, Kadir Öztürk, Mesut Yılmaz, Mahmut Gökdağ, Murat Ayten

**Affiliations:** ^1^Neurospinal Academy, Neurosurgery, 34940 Istanbul, Turkey; ^2^Tepecik Education and Training Hospital, Clinic of Neurosurgery, 35120 Izmir, Turkey

## Abstract

*Objective*. Many surgical approaches have been defined and implemented in the last few decades for thoracic disc herniations. The endoscopic foraminal approach in foraminal, lateral, and far lateral disc hernias is a contemporary minimal invasive approach. This study was performed to show that the approach is possible using the microscope without an endoscope, and even the intervention on the discs within the spinal canal is possible by having access through the foramen. *Methods.* Forty-two cases with disc hernias in the medial of the pedicle were included in this study; surgeries were performed with transforaminal approach and microsurgically. Extraforaminal disc hernias were not included in the study. Access was made through the Kambin triangle, foramen was enlarged, and spinal canal was entered. *Results.* The procedure took 65 minutes in the average, and the mean bleeding amount was about 100cc. They were mobilized within the same day postoperatively. No complications were seen. Follow-up periods range between 5 and 84 months, and the mean follow-up period is 30.2 months. *Conclusion.* Transforaminal microdiscectomy is a method that can be performed in any clinic with standard spinal surgery equipment. It does not require additional equipment or high costs.

## 1. Introduction

Symptomatic thoracic disc herniation is one of the rare degenerative diseases of the spine. Its share among other similar pathologies can be indicated as 0,25 to 1%. Studies conducted on the general population revealed its incidence rate as approximately 1/1000000 patient in one year [1–3]. This rate applies to both women and men, and it is usually observed at ages 30 to 50 [4]. The pathology usually localizes at the medial or mediolateral region and rarely can one see a real lateral localization of the pathology [3, 5]. The rate of incidence for calcified pathologies is 30 to 70% [6, 7].

Decision for the surgical indication is controversial, due to the limited amount of information obtained so far on the natural course of thoracic disc herniation [8, 9]. On the one hand, the necessity of surgical treatment is not a matter of debate in the presence of progressive myelopathy symptoms, but on the other, it is still not clear whether the surgery can fix the symptoms in patients presenting radicular pain.

Wood et al. followed up 20 patients, who were randomly diagnosed with thoracic disc pathology, for an average duration of 26 months and reported that the patients were still asymptomatic at the end of this follow-up period [10]. Brown et al. assessed 55 symptomatic patients with thoracic disc pathology and reported that 77% of the 40 patients (73%) who were given nonsurgical treatment had complete recovery from their symptoms [11].

Although the decision for the eligible surgical approach is still controversial, the search is ongoing to find an effective, safe, and simple surgical approach especially for thoracic disc pathologies with medial localization.

## 2. Material and Method

Forty-two cases with disc hernias in the medial of the pedicle and foraminal disc hernias were included in this study and surgeries were performed with transforaminal approach and microsurgically. Extraforaminal disc hernias were not included in the study. Access was established with the patient in flexed prone position through an incision of 2–2.5 cm in length made 6 to 10 cm away from the midline (mean 8 cm). After opening the fascia, digital dissection was used to advance in the intermuscular space to expose the transverse process and the lateral of the superior articular process (lateral of the facet joint junction). The planned disc level was accessed after the control of the distance with scopy. Access was made through the Kambin triangle, foramen was enlarged, and spinal canal was entered (Figure 2). Transforaminal microdiscectomy (TFMD) was performed using standard instruments.

### 2.1. Surgical Technique

The materials we use in this procedure are those available in any center where microneurosurgery is performed: surgical microscope, radiolucent operation table, C-arm scopy, microsurgical instruments, Landolt separators used in pituitary surgery, Meyerding separators used in lumbar microdiscectomy, separators used in anterior cervical approach (Caspar, Clovard, etc.), or nasal speculum whichever is found or convenient.

We perform the procedure with patient in prone position under spinal or general anesthesia. The table can be tilted to the lateral. The level is determined using C-arm scopy and AP and lateral scopy. Later, depending on the anatomy of the area, type of the pathology, and depth of the pathology, a skin incision of 2–2.5 cm in length is made at 6 to 10 cm lateral of the midline (Figure 3). After cutting the fascia, access will be with digital dissection between the paraspinal muscles and the lateral side of the facet and transverse processes and the intertransverse ligament. Following the repeat scopy control, the separator is placed and the required distance is reached. The disc is reached directly from the inferior of the foramen if the disc has no cranial or caudal extensions. Dissection is started on the transverse process-pedicle junction in the superior of the foramen. The root is exposed first, and then discectomy is performed. The pedicle of the lower vertebra prevents exploration in discs with caudal extension.

## 3. Findings

5 of the cases were males, while 10 were females. Ages ranged between 20 and 62 (average 44.3). There was thoracal (Th) 4-5 disc hernia in 1 case, Th (6-7) in 1 case, Th (8-9) in 1 case, Th (9-10) in 3 cases, Th (10-11) in 4 cases, Th11-12 in 4 cases, and Th (12)-Lumbar (L)1 in 1 case.

They were mobilized within the same day postoperatively and were discharged the next day. No complications were seen except for mild radicular paresthesia in 1 case that lasted for about 8 weeks. Follow-up periods ranged between 10 and 72 months, and the mean follow-up period is 34.8 months.

Preoperative pain score in cases was changing between 5 and 8 (mean 6) according to VAS (Visual Analogue Scale). Pain score was marked between 0 and 1 (mean 0.87) by the patients, according to VAS, postoperatively.

At ODI (Oswestry Disability Index) questioned form that was filled preoperatively, score was between 46% to 90% (mean 72.27%) (daily life completely restricted because of pain), and postoperatively it was 0% to 64% (mean 18%) (pain is not a serious problem in daily life).

Compared with preoperative results, postoperative VAS and ODI results have significant improvement (*P* < 0.001). Patients' pathology levels, preoperative and postoperative VAS, ODI, and neurological statues are summarized in Table 1.

41 of patients answered “Yes” when 1 patient answered “Undecided, maybe” to the question “If you knew the result before, would you have taken this treatment anyway?” at a postoperatively filled patient satisfaction form.

## 4. Sample Cases

**Figure 1:**
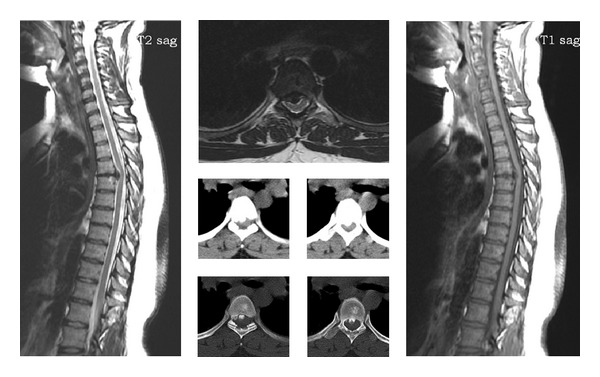
35-year-old female. Back pain and also in both legs. Progressive weakness in lower extremities. Preoperative VAS was 5. In the neurological examination there was paraparesis in low extremities (Case 1). Preoperative views of the patient revealed a thoracic 4-5 disc herniation.

**Figure 2:**
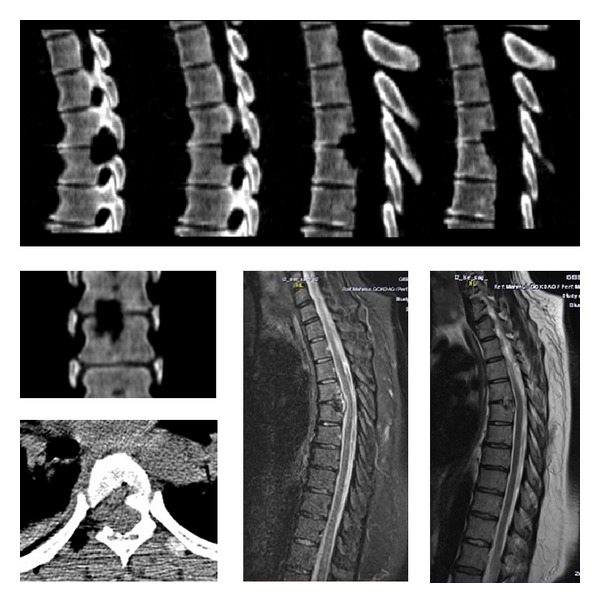
Early postoperative images of the patient after the performance of right transforaminal approach (Case 1).

**Figure 3:**
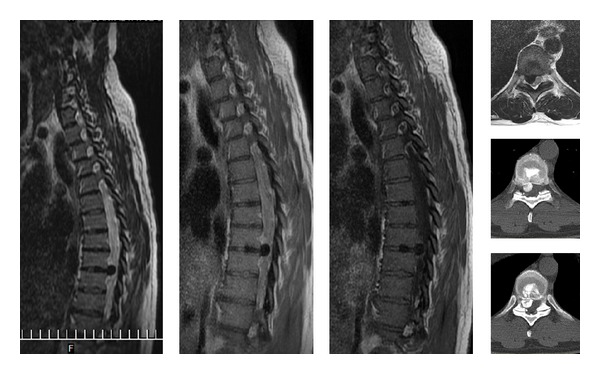
36-year-old female. Weakness in lower extremities. Preoperative ASIA was C (Case 5). Preoperative CT and MRI revealed a thoracic 8-9 disc herniation.

**Figure 4:**
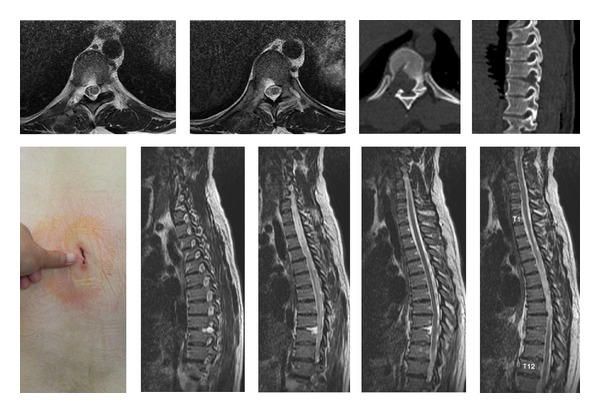
Postoperative CT, MRI images of Case 5. View of the incision.

**Figure 5:**
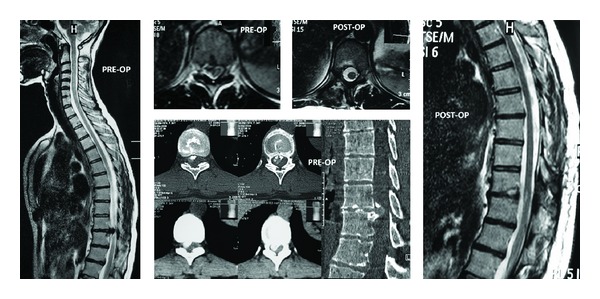
34-year-old female. In the neurological examination there was paraparesis in lower extremities (ASIA C). Cord compression of a thoracic 9-10 disc herniation (Case 10). Preoperative CT and MR images at the left side and postoperative images at the right side.

See Figures 1, 2, 3, 4, and 5.

## 5. Discussion

Indications of thoracic disc herniation and the surgical method of selection have long been under discussion. There are no absolute factors to help one take a decision on the surgical treatment, as the clinical natural course of thoracic disc herniation is still not fully discovered. Many surgical approaches have been defined and implemented in the last few decades. The best method for thoracic disc herniation is still controversial. Except for the laminectomy method that has been abandoned lately, a comparison of the results obtained by studies on various surgical methods indicates that 60 to 80% of the patients recover from the pain or improve their neurological picture.

Posterior laminectomy and/or discectomy is the first method used in surgical treatment of thoracic disc herniation [12]. By using this method, it is difficult to decompress midline disc pathologies attached to the dura. The risk of morbidity is high, and even paraplegia may develop. Furthermore, it contains the risk of late kyphotic deformity development [13, 14]. This method has now become historic, and it is not anymore used as a surgical treatment approach for thoracic disc herniation [15].

Transpedicular approach, transfacet pedicle sparing approach, costotransversectomy, and transfacet/transforaminal approach are listed among posterolateral approaches [16–23].

Perot Jr. and Munro [14] described the transthoracic approach in 1969 and in 1988 Bohlman and Zdeblick recapitulated this approach. This technique provides access to all levels under T4. It provides direct visibility in central, paracentral, and lateral pathologies [24]. The method proves to be effective in soft and hard pathologies, and it has high efficacy in multilevel pathologies [25]. The method presents high rates of complications such as atelectasis, pleural effusion, and pneumonia, which is a disadvantage. If the surgeon has to free the diaphragm, hernia may develop. Large arteries or venous structures may be damaged, and left-side approaches bear the risk of infarct and impaired blood supply to the spinal cord due to the obstruction of Adamkiewicz artery. However, Mulier and Debois indicated that even though pulmonary complications may be observed unlike lateral and posterolateral approaches, this approach yielded better neurological improvement [26]. Otani et al. described transthoracic extrapleural approach to reduce the risk of pulmonary complications [27].

The advantages of anterior video-assisted thoracoscopic approach include minimal dissection, low morbidity, no need to retract for rib resection, short hospital stay, and short rehabilitation period. The biggest disadvantage is that the surgeon should be particularly trained to perform this approach. In their study involving 29 patients, Regan et al. reported 76% satisfactory results [25].

Transforaminal endoscopic discectomy is among the methods applicable for thoracic disc disease. It may be used not only for far lateral and foraminal discs but also in midline discs [28]. Transforaminal endoscopic discectomy (TFD) has increased success rates in eligible patients. Computed Tomography helps to discover the bone structure at the preoperative stage.

Transforaminal microdiscectomy (TFMD) saved the surgeons from the two-dimensional limitation of endoscopy and offered them a three-dimensional view. Compared to classical surgery, TFMD reduced the rate of instability and muscle denervation. Early postoperative mobilization of the patient and short hospital stay are the other advantages of this system. It offers a safer surgery by providing better microscopic view and light, which neurosurgeons are more accustomed to. Furthermore, TFMD does not require additional equipment, which is a cost-reducing factor.

## 6. Conclusion

Transforaminal microdiscectomy can be performed by using standard neurosurgery equipment and it does not require additional surgical equipment. TFMD can be performed without causing neurologic deficits and wide decompressions leading to instability.

## Figures and Tables

**Table 1:** Preoperative and postoperative features of the patients.

Cases	Gender/age	Level	Preop. VAS	Preop. ODI	Preop. ASIA	Side	Time	Postop. VAS	Postop. ODI	Postop. ASIA
Case 1	F/35	T4-T5	5	82	C	Right	135	0	0	E
Case 2	M/52	T6-T7	5	66	D	Left	130	1	24	D
Case 3	F/54	T8-T9	6	86	C	Right	90	1	22	D
Case 4	M/57	T9-10	6	86	C	Right	120	1	26	D
Case 5	F/36	T9-T10	6	80	C	Right	115	0	0	E
Case 6	F/46	T9-T10	6	68	D	Right	95	1	8	E
Case 7	F/48	T10-T11	5	64	D	Right	85	1	14	E
Case 8	M/62	T10-T11	6	90	C	Left	105	2	64	C
Case 9	F/55	T10-T11	6	86	C	Right	85	1	34	D
Case 10	F/34	T10-T11	6	84	C	Right	130	2	26	D
Case 11	F/45	T11-T12	5	68	D	Left	110	1	14	E
Case 12	F/40	T11-T12	7	66	D	Right	95	1	28	D
Case 13	F/56	T11-T12	6	62	D	Left	100	1	10	E
Case 14	M/20	T11-T12	7	50	E	Right	90	0	0	E
Case 15	M/25	T12-L1	8	46	Ê	Left	90	0	0	E
